# Supplementary material for: Complexome profiling of the Chlamydomonas psb28 mutant reveals TEF5 as an early PSII assembly factor
Source: Plant Cell. 2025 Mar 17;37(6):koaf055. doi: 10.1093/plcell/koaf055 (PMC12164590; doi:10.1093/plcell/koaf055)
Supplement: koaf055_Supplementary_Data [file koaf055_supplementary_data.zip › psb28_interactive.zip!/plots/intensity/qualityPlots/peptides/CGL71_Cre12.g524300.t1.2.html]

### Cre12.g524300.t1.2

Trivial name: CGL71  
SeqCoverage [median]: 11.60  
Mass: 28.37 kDa  
  
 

| Sequence | MissedCleavage | Mass [Da] | wtIntensity | muIntensity | Score |
| --- | --- | --- | --- | --- | --- |
| ELDEGAK | 0 | 760.36 | 2558.83 | 6600.97 | 110.61 |
| ELDEGAKGLGER | 1 | 1272.63 | 6449.14 | 5818.00 | 90.73 |
| KKLFTQATK | 2 | 1063.64 | 1254.36 | 1011.20 | 151.22 |
| LFTQATK | 0 | 807.45 | 8593.66 | 11053.91 | 129.56 |
| TDMAIEEYR | 0 | 1126.50 | 1705.74 | 6097.33 | 183.65 |
|  |  |  | 20561.7 | 30581.4 |  |
